# The preventive effect of the bounding exercise programme on hamstring injuries in amateur soccer players: the design of a randomized controlled trial

**DOI:** 10.1186/s12891-017-1716-9

**Published:** 2017-08-22

**Authors:** S. Van de Hoef, B. M. A. Huisstede, M. S. Brink, N. de Vries, E. A. Goedhart, F. J. G. Backx

**Affiliations:** 1 Department of Rehabilitation, Physical Therapy Science and Sports, Rudolf Magnus Institute of Neurosciences, University Medical Center Utrecht, Utrecht, The Netherlands; 2 Center for Human Movement Sciences, University of Groningen, University Medical Center Groningen, Groningen, The Netherlands; 3 FIFA Medical Center, Royal Netherlands Football Association, Zeist, The Netherlands

**Keywords:** Injury, Prevention, Plyometric exercise, Football

## Abstract

**Background:**

Hamstring injuries are the most common muscle injury in amateur and professional soccer. Most hamstring injuries occur in the late swing phase, when the hamstring undergoes a stretch-shortening cycle and the hamstring does a significant amount of eccentric work. The incidence of these injuries has not decreased despite there being effective injury prevention programmes focusing on improving eccentric hamstring strength. As this might be because of poor compliance, a more functional injury prevention exercise programme that focuses on the stretch-shortening cycle might facilitate compliance. In this study, a bounding exercise programme consisting of functional plyometric exercises is being evaluated.

**Methods:**

A cluster-randomized controlled trial (RCT). Male amateur soccer teams (players aged 18–45 years) have been randomly allocated to intervention and control groups. Both groups are continuing regular soccer training and the intervention group is additionally performing a 12-week bounding exercise programme (BEP), consisting of a gradual build up and maintenance programme for the entire soccer season. The primary outcome is hamstring injury incidence. Secondary outcome is compliance with the BEP during the soccer season and 3 months thereafter.

**Discussion:**

Despite effective hamstring injury prevention programmes, the incidence of these injuries remains high in soccer. As poor compliance with these programmes may be an issue, a new plyometric exercise programme may encourage long-term compliance and is expected to enhance sprinting and jumping performance besides preventing hamstring injuries.

**Trial registration:**

NTR6129. Retrospectively registered on 1 November 2016.

## Background

Thirty-one percent of all injuries in male soccer players are muscle injuries, which account for 27% of injury-related time off soccer [1, 2], and 37% of all soccer-related muscle injuries are hamstring injuries. These injuries can be classified as high-speed running injuries and stretching injuries [1, 3, 4], with the sprint-type injury being the most common type of hamstring injury in soccer [2–5]. Hamstring injuries also have the highest re-injury rate and are associated with the longest time off play and training (>28 days) [1, 2, 6]. Although several intrinsic and extrinsic risk factors have been identified, to date the incidence of hamstring injuries has not diminished [6–11].

Sprint-type hamstring injuries mostly occur in the late swing phase [4, 5, 12, 13], when the hamstring muscles are maximally stretched during limb deceleration [4, 5, 13–16]. In the second half of the swing, the bi-articular hamstring muscles undergo a stretch-shortening cycle [14]. The greatest stretch is seen in the femoral biceps, which is the hamstring muscle most often injured [3, 5, 13, 14]. The stretched hamstring muscle actively contracts in the swing phase, which could indicate that the hamstrings contract eccentrically in the late swing phase [13]. In addition, it has been found that as the sprint speed increases, so too does the amount of stretch and negative work of the hamstring muscles [13]. Thus it has been suggested that increasing eccentric strength might reduce the risk of sprint-type hamstring injury.

Recent studies have shown that improving eccentric hamstring strength by means of the Nordic Hamstring Exercise (NHE) can reduce the incidence of hamstring injuries by 66–70% [16, 17]. Yet the incidence of hamstring injuries has not only not decreased in the past 10 years, but has actually increased by 4% [18]. This might be because these exercises are not performed correctly or because compliance is poor. A recent study among Champions league and Norwegian premier league teams confirmed this hypothesis: only 6% of the participating teams performed the NHE according the prescribed programme [19]. The same trend has been seen in Dutch soccer players [20], with most teams no longer performing the prevention programme 3 years after its introduction [20]. Arguments for non-compliance mentioned in the Netherlands are lack of time, delayed onset muscle soreness, the need to sit on the ground or a mat, and not sport- specific enough to incorporate into the warming-up [20].

The bounding exercise programme (BEP) is another potentially effective training programme that can be done after warming-up. It consists of single leg jump exercises characterized by a stretch-shortening cycle: eccentric pre-stretch phase, amortization phase, and concentric shortening phase [21]. This stretch-shortening cycle strengthens the elastic properties of connective tissue, thereby improving (eccentric and concentric) strength and power by allowing the muscle to accumulate (pre-stretch / eccentric phase) and release (concentric phase) energy [22, 23]. Specific physiological adaptations induced by plyometric training are increased motor unit activation, increased passive tension of the muscle- tendon complex, and improvement of cross-bridge mechanics [24, 25]. These adaptations are associated with improved strength, increased joint stiffness, and improved neuromuscular control and functional performance [26–28]. Plyometric training is already used widely in intermittent team sports in order to enhance sprinting and jumping performance [22, 29–35] and might reduce sprint-type hamstring injuries.

In summary, hamstring injuries are a major health problem in soccer and their incidence has increased over recent years, despite there being effective prevention programmes. This is possibly because of poor compliance. There is an urgent need for a functional, short, easy-to-implement, sport-specific hamstring injury prevention programme that includes eccentric and plyometric exercises, so as to improve the performance of sport-specific tasks, such as sprinting and jumping, and which is likely to be adopted. The BEP could fulfil this need. The aim of this hamstring injury prevention study-3 (HIPS-3) is to investigate the preventive effect of a BEP on hamstring injuries in male amateur soccer players. The secondary aim is to investigate compliance with this programme during the soccer season 2016–2017 and at the start of the soccer season 2017–2018.

## Methods/design

### Design and study setting

This prospective, cluster-randomized trial was designed in accordance with the guidelines of the Standard Protocol Items: Recommendations For Interventional Trials (SPIRIT) [36]. The study is being carried out in cooperation with the University Medical Centre of Utrecht and the Royal Netherlands Football Association (KNVB) in Zeist (the Netherlands). As the BEP is being investigated in a real-life setting, soccer teams from four different districts, playing in Dutch first-class amateur field soccer competition, were invited to participate in this study. These teams usually play one (or two) matches a week, with 2–3 training sessions per week. Each district has two competitions at first-class level.

### Recruitment of participants and randomization

Dutch male soccer players (age 18–40 years) playing in the Dutch first-class amateur competition were eligible for participation. All players were asked to give their written informed consent before the start of the study. Players who did not give their informed consent, who joined a team after the start of the study, or who had a lower than average understanding of Dutch were excluded.

After selection of the four districts, a top-down strategy was carried out to recruit the soccer teams. First, the boards of the clubs were informed about the study by means of an email from the director of amateur soccer of the KNVB and the research team. Then the members of the medical staff and the coaches were invited, by telephone, to attend a meeting to tell them about the purpose and methods of this study. If the clubs decided to participate in this study, all players of the participating teams received an information letter including an informed consent form. To avoid a risk of contamination, the teams were randomized as clusters instead of individual players [37]. Randomization was done independently by an online randomizer (https://www.randomizer.org/) and an equal number of teams were assigned to the intervention and control groups. After randomization, meetings were organized in each district to inform staff and players in the intervention group about the BEP.

### Intervention

The intervention group is performing the BEP during the entire 2016–2017 outdoor soccer season. In the first 12 weeks, there is a gradual build up of walking lunges, triplings and drop lunges, and bounding (alternating leg jumps) (Table 1).

**Table 1:** BEP Program

Week	Programme
1	2x30m walking lunges (2 × 10)
2	3x30m walking lunges (3 × 10)
3	3x30m walking lunges +1x30m triplings + droplunges)
4	2x30m triplings + droplunges (2 × 10)
5	3x30m triplings + droplunges (3 × 10)
6	3x30m triplings + droplunges +1 × 30 m bounding
7	2x20m bounding (+/− 7 jumps)
8	3x20m bounding (+/− 7 jumps)
9	4x20m bounding (+/− 7 jumps)
10	3x30m bounding (+/−10 jumps)
11	4x30m bounding (+/− 10 jumps)
12	4x30m bounding (in the least possible jumps)
13 untill end of competition	3x30m bounding (in the least possible jumps)

This gradual build-up from basic concentric strength exercise to eccentric strength exercise followed by plyometric exercise should improve functional movement patterns and increase strength [38]. All exercises are performed over a distance of 30 m in accordance with the SCORE project, which uses another form of bounding, focusing on vertical stability [39]. After the build-up phase, participants continue with the BEP during warming up in two training sessions and follow a prescribed intensity training programme. After the winter break, when there is no structural training, teams will restart the BEP. In order to compensate for potential de-training effects [40–42], starting with the same programme as performed in week 6. If a player is injured at the start of the programme and recovers from this injury, he re-starts the BEP programme. If a player is injured in weeks 1–6 and achieves full recovery within 4 weeks, he re-starts the BEP at −1 week (i.e. a player injured in week 4 and who achieves full recovery within 4 weeks re-starts the BEP in week 3). If a player is injured in weeks 7–13 and achieves full recovery within 4 weeks, he re-starts the BEP in week 6. If an injury last longer than 4 weeks, the player is asked to contact the research team before resuming the BEP (Table 2).

**Table 2:** Restarting BEP after injury

Week nr. BEP of Injury occurrence	Period of injury (weeks)	Restarting BEP
1–6	<4	Week injury occurrence −1
1–6	>4	Contact with research team
7–13	<4	Week 6
7–13	>4	Contact with research team

#### Walking lunges (Fig. 1)

The player starts in a standing upright position and steps forward with the right leg. From this position, the left knee moves downwards to the ground while the upper body remains upright. This movement is followed by a step forward passing the right foot. From this second position, the right knee moves downwards to the ground, followed by a step forward passing the left foot, etc. It is important that the flexion of the leading knee does not exceed 90 degrees, and that the shoulder, hip, and knee of the back leg are in a vertical line [43].

**Fig. 1:**
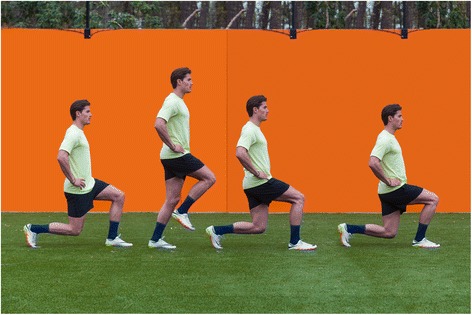
Walking lunges

#### Triplings followed by drop lunges (Fig. 2)

Triplings are a classic running exercise. The player starts in a standing position and initiates a fast small step forward and lands on his forefoot, followed by the other leg. This sequence is repeated 4 times. At the same time, the player performs a fast arm swing, After 4 repetitions, the player jumps vertically jump and lands in the end-position of the lunge [43].

**Fig. 2:**
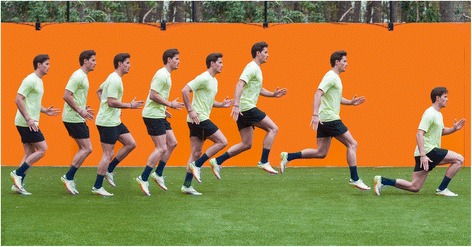
Triplings followed by drop lunges

#### Bounding (Fig. 3)

The bounding exercise, in the literature referred to as ‘a running bound’ or ‘alternate leg bounding’, is a popular running-specific exercise that can be categorized as plyometric exercise. These plyometric exercises contain three phases: the eccentric pre-stretch, the amortization phase (time between eccentric and concentric contraction), and the concentric shortening phase [21]. The player starts running and after 3 m he performs horizontal jumps (bounding). The focus of this exercise is to achieve horizontal position of the femur in swing-phase, a full foot landing, and as fast as possible horizontal speed. The running start is chosen to reach a maximum speed in bounding by getting a maximum hamstring musculo-tendon stretch and force [13]. All players have to cover 30 m excluding the 3 m running start.

**Fig. 3:**
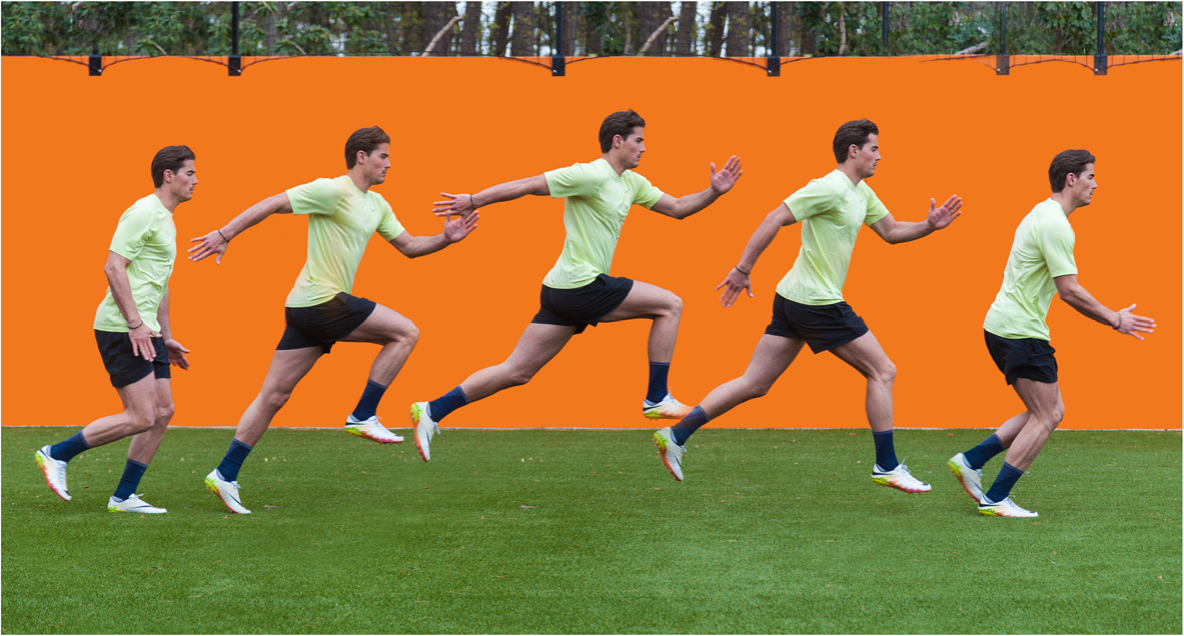
Bounding

### Implementation procedures

The medical staff and/or coaches of the intervention teams were instructed how to implement the BEP during the above-mentioned instruction meetings. During these meetings, there was a video presentation of the BEP programme, followed by a live demonstration on the soccer field. Emphasis was on key aspects of the intervention and the performance of the exercises. All coaches and medical staff received the instruction-video and instructions by email and in writing.

The research team is always accessible to answer questions by telephone or email. All participating teams are visited, and the medical staff has been asked to record the intervention on camera (cell phone) and to send the recording to the research team for analysis and feedback, at least every 3 weeks in the build-up phase. The BEP is performed at the end of the warming-up. Coaches and medical staff were advised to allow a short active recovery period before the rest of the training session if players complain of tired legs.

### Data collection

#### Baseline characteristics

All participants were asked to complete a questionnaire on baseline characteristics, namely, date of birth, nationality, height, weight, years of experience as a soccer player, field position, leg dominance, preventive measures for hamstring injuries, preventive measures for other injuries, study load/ workload and capacity, hours of sleep, hours of travel, current state of the hamstring muscles in relation to activities, and injury history.

#### Exposure, injuries, and compliance

Exposure, injuries, and compliance are self-reported weekly throughout the entire soccer season (39 weeks), with players being allowed to choose whether they prefer to register by SMS or by e-mail (NetQ). Each Monday, all players receive four short questions regarding how long they have trained (minutes), how long they have played in a match (minutes), and whether they have had a hamstring injury or another’ injury. The players in the intervention group receive a fifth question regarding the amount of BEP they have performed in the past week.

Injuries are divided into hamstring injuries, other injuries, and recurrent (hamstring) injuries. A hamstring injury is defined as any physical complaint affecting the posterior side of the upper leg resulting in an inability to play or train regardless of the need for medical attention or time off soccer-associated activities [44]. In the case of a hamstring injury, both the player and the medical staff separately fill in a hamstring injury questionnaire to record epidemiology, severity, and aetiology of the injury. Other injuries are defined as any physical complaint, other than affecting the posterior side of the upper leg, that results in an ability to play or train regardless of the need for medical attention or time off soccer-associated activities [44]. Other injuries are recorded for measuring exposure and are not further specified by an injury follow-up questionnaire. Recurrent injuries are defined as an injury of the same type and at the same site as an index injury occurring after a player has returned to full play/training. In the case of a recurrent hamstring injury, the player and medical staff will receive the same hamstring injury questionnaire as for the primary hamstring injury.

Exposure (in minutes) is divided into match play and training sessions. Training exposure is defined as team-based and individual physical activities under the guidance of the team’s coaching or fitness staff that are intended to maintain or improve players’ soccer skills or physical condition [44]. Match play is defined as a play between two different teams [44].

Intervention compliance is measured weekly by SMS or email (NetQ). The number of metres of the BEP performed is registered as metres/week. Compliance is expressed as a percentage by dividing the meters BEP performed by the metres mentioned in the programme. At the end of the study period, overall compliance will be investigated by means of a self-administered questionnaire.

**Fig. 4:**
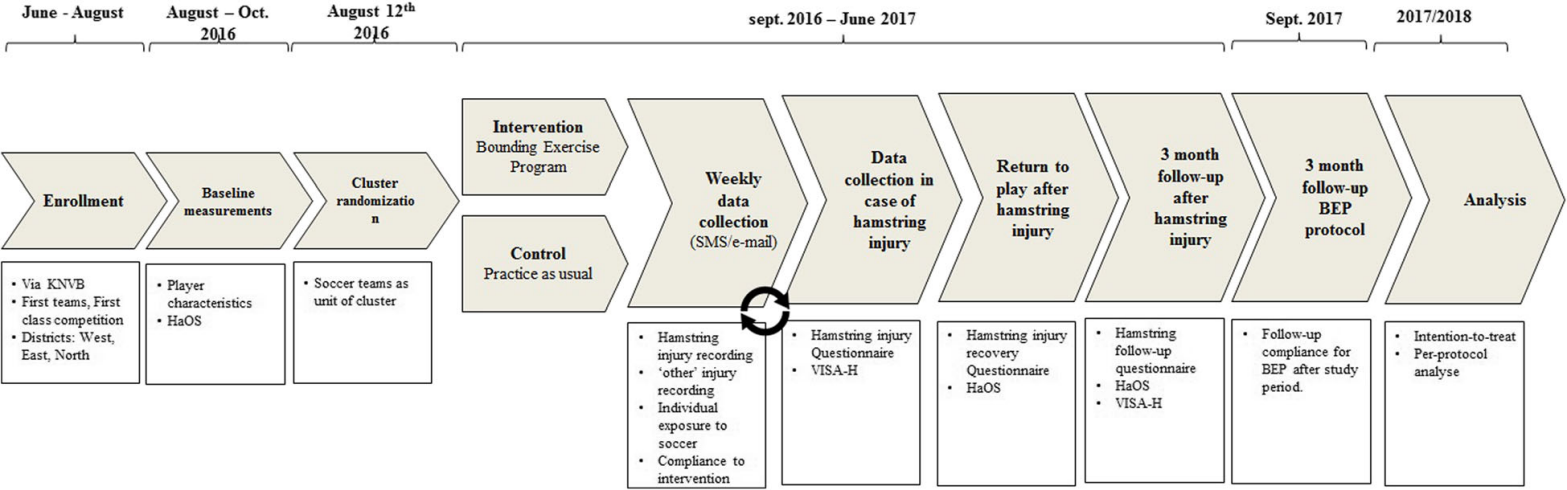
Study procedure

**Fig. 5:**
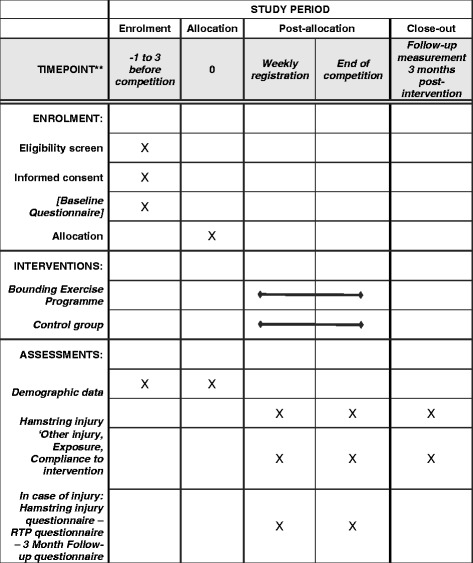
SPIRIT figure

Long-term compliance with the BEP will be measured once, at the start of the new soccer season 2017–2018. All players in the intervention group will be asked how many metres BEP they performed in that week and how many days ago their last bounding session was (Figs. 4 and 5).

### Outcomes

The primary outcomes are the incidence and severity of hamstring injuries. The secondary outcome is compliance with the BEP. The hamstring injury incidence is recorded per 1000 h of exposure, and severity is operationalized as the number of days since the day of injury and the day of full return to play [17]. Compliance is expressed as a percentage of BEP completion.

### Sample size

The sample size was calculated based on previous studies. Earlier findings showed a reduction in hamstring injury incidence of up to 70% with eccentric training of the hamstring muscle, an injury incidence of 1 in 11 players per soccer season, and recurrence rates of 12–30% [16, 17]. On the basis of these findings and two-sided testing, significance level of 0.05, power 0.8, an inflation correction for cluster randomization of 1.19, drop-out of 20%, and loss to follow-up of 20%, we calculated that 26 teams of approximately 16 players would have to be included. Taking into account that the follow-up period is in a new soccer competition, with a larger loss to follow-up, we included 10% extra players, so that in total the study includes 30 teams with about 16 players/team (*n* = 480).

### Statistical methods

SPSS version 22 will be used to analyse the data. Descriptive statistics will be used to analyse the baseline characteristics. The incidence of hamstring injuries will be analysed on an intention-to-treat basis. The primary outcomes will be compared in the intervention and control groups using Chi square tests, Poisson general log-linear analysis, and Cox regression analysis with survival curves.

### Ethical approval

This trial was approved by the Medical Ethics committee of University Medical Centre of Utrecht (16–332\C) and is registered in the Dutch trial register (http://www.trialregister.nl/trialreg/index.asp) (NTR6129). All participants received a written information letter, and any questions were answered (verbally or in writing) by one of the researchers (SvdH). All participants were asked for their informed consent, and all data are handled according to legal requirements.

## Discussion

Hamstring injuries are a major problem in amateur soccer players. Recent studies have shown that a reduction in hamstring injury incidence up to 70% can be achieved by eccentric training following the NHE programme [16, 17]. While NHE compliance was high in studies, the NHE programme has not been adopted in amateur soccer and the number of hamstring injuries has not diminished yet [17, 18]. Arguments for non-compliance are lack of time, delayed onset muscle soreness, the need to sit on the ground or a mat, and not sport- specific enough to incorporate into the warming-up [19, 20].

The BEP, consisting of a gradual build-up to plyometric (bounding) exercises, is a more sport-specific injury prevention programme, so that compliance is expected to be better. It can be easily implemented during warming-and is expected to improve sprint and jumping performance [29–32].

This study has the advantage of a standardized methodology and consensus regarding definitions of injuries and exposure [44]. The self-reported registration via modern systems, such as SMS and NetQ, will probably ensure valid injury registration and optimal compliance.

## Abbreviations

BEP: Bounding Exercise Programma
HIPS-3: Hamstring Injury Prevention Study-3
KNVB: Royal Netherlands Football Association
NHE: Nordic Hamstring Exercise
SPIRIT: Standard Protocol Items Recommendations For Interventional Trials
